# Small Intestinal Bacterial Overgrowth in Patients with Roux-en-Y Gastric Bypass and One-Anastomosis Gastric Bypass

**DOI:** 10.1007/s11695-022-06299-z

**Published:** 2022-10-05

**Authors:** Urška Novljan, Tadeja Pintar

**Affiliations:** 1 Medical Faculty Ljubljana, University Medical Centre Ljubljana, Ljubljana, Slovenia; 2 Abdominal Surgery Department, University Medical Centre Ljubljana, Ljubljana, Slovenia; 3 University of Ljubljana, Medical Faculty, Ljubljana, Slovenia

**Keywords:** Small intestinal bacterial overgrowth (SIBO), Obesity, Bariatric surgery, Roux-en-Y gastric bypass, One-anastomosis gastric bypass, Glucose breath test, Liver injury

## Abstract

**Background:**

Small intestinal bacterial overgrowth (SIBO) is defined as an excessive growth and/or changed composition of bacteria in the small bowel. Obese patients are at increased risk of SIBO and related complications. The purpose of this study is to evaluate the incidence of SIBO after bariatric bypass procedures, connection between SIBO, symptoms, comorbidities, and liver pathology.

**Methods:**

Patients underwent a hydrogen breath test with glucose substrate (25 g/200 ml of water). The demographic, anthropometric data, comorbidities, and symptoms were analysed with a questionnaire. In 45 patients, the NAFLD Activity Score was evaluated in liver biopsies.

**Results:**

Glucose breath test was positive in 24/56 (43%) of patients and was associated with higher frequency of defecation (*p* = 0.022), lactose intolerance (*p* = 0.047), scleroderma (*p* = 0.042), irritable bowel syndrome (*p* = 0.018), and diabetes (*p* = 0.002). Mean NAFLD Activity Score in SIBO patients (*n* = 18) was 3.33 and 3.00 in non-SIBO patients (*n* = 27). In SIBO-positive cohort of patients, a statistically important trend in difference between NAS and difference to range value anti-Xa 4 h after subtherapeutic dose application was calculated.

**Conclusions:**

The incidence of SIBO after bariatric surgery bypass procedures is alarmingly high (43%). The results of our study conclude that diagnosis cannot be set based on specific symptom and SIBO is related to reduced response to the application of LMWH. Mandatory SIBO screening and appropriate treatment would affect the clinical outcome of the underlying disease, improve it significantly, and prevent the development of its complications.

**Graphical abstract:**

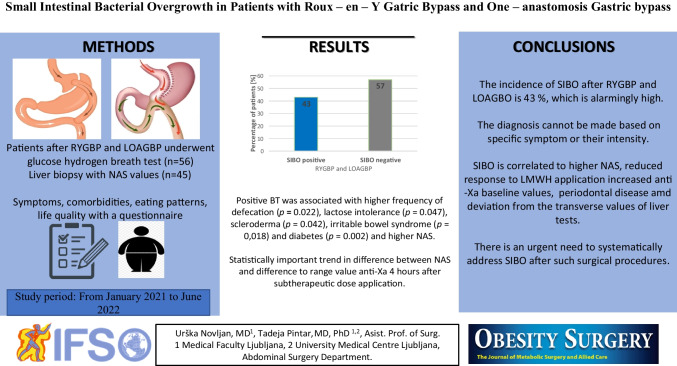

## Introduction

Obesity is a worldwide public health problem continuing to rise rapidly [1]. The incidence of different diseases, such as cardiovascular disease, type 2 diabetes, and cancer, has been strongly associated with obesity, leading to a significant economic and social burden of pandemic magnitudes [1, 2]. Obesity is considered as a complex and multifactorial disease, and one of the astonishing findings over the past decade has been the association and causative role played by gut bacteria in the pathophysiology of obesity [1, 3] and obesity-related complications and importantly, including a significant worsening of obesity-related diseases [3].

Small intestinal bacterial overgrowth (SIBO) is defined as a heterogeneous pathology characterised by an increased number and/or abnormal type of bacteria in the small intestine responsible for digestive symptoms such as bloating, abdominal pain, nausea, or diarrhoea [4, 5]. The recognition of SIBO is prudent, as the condition has been associated with altered small intestinal motility, fat malabsorption, vitamin deficiencies, reversible protein-losing enteropathy, and malnutrition [6, 7].

Several data suggest that SIBO could play a role in the pathophysiology of metabolic-associated fatty liver disease (MAFLD), formerly known as non-alcoholic fatty liver disease (NAFLD) [8, 9]. Because of the increasing prevalence of obesity, NAFLD has become one of the major causes of liver diseases [10]. The spectrum of liver injury is broad, ranging from pure steatosis to non-alcoholic steatohepatitis, a more severe form of NAFLD, which can progress to cirrhosis, liver failure, and hepatocellular carcinoma (HCC) [8, 11]. Microbiota pharmacological modulation seems to be a promising tool for a new therapeutic approach to NAFLD and in prevention of its complications [12].

The only effective treatment considered for sustained long-term weight loss is bariatric surgery [2], with commonly performed procedures Roux-en-Y gastric bypass (RYGBP) and one-anastomosis gastric bypass (OAGBP). Despite improving the overall quality of life of individuals, bariatric surgery is connected to the development of gastrointestinal symptoms, subsequently interfering with post-surgical quality of life and patient satisfaction [6, 13]. The modification of normal gut anatomy after bariatric surgery may induce bacterial stasis and subsequently precipitate SIBO and nutritional deficiencies [2, 6].

The present study sought to identify the incidence of SIBO after RYGBP and laparoscopic OAGBP (LOAGBP), connection between SIBO and certain symptoms, comorbidities, and related liver pathology and correlation SIBO to low molecular weight heparin (LMWH) application to help prompt early evaluation of this condition in this patient population.

## Methods

### Study Design

An observatory randomised analytical cross-sectional study was performed at University Medical Centre Ljubljana between January 2021 and June 2022. All of 56 included patients systematically had a glucose hydrogen (H_2_) breath test (BT) and filled out a questionnaire. Forty-five included participants had liver biopsy and were tested for subtherapeutic LMWH application related to SIBO status.

### Participants

The inclusion criteria were adults which had RYGBP or LOAGBP between January 2017 and June 2022, with or without gastrointestinal symptoms, who signed the free consent form. The exclusion criteria were incapacity of preparation or execution of the study; inability of finishing the study; use of antibiotics, prokinetics, and/or laxatives in the past 2 weeks; and basal value of hydrogen > 10 ppm in two different measures, 20 min apart.

### Glucose Hydrogen Breath Test

Participants were instructed to ingest a low-fermentation diet 24 h before the exam and avoid smoking and physical activity on the day of the exam. Subjects fasted overnight (12 h) and during the H_2_ BT. At the start of the test, a basal sample of expired air was collected by means of an H_2_ BT device (Lactofan 2 Fischer®, Leipzig, Germany). The results were expressed as parts per million (ppm). If the first measure of H_2_ was < 10 ppm, the participants ingested 25 g of glucose diluted in 200 mL of water. Every 20 min, in total, 120 min 6 expired air samples were collected. An elevation of more than 12 ppm according to the basal value within 120 min was deemed to be a positive result, indicating SIBO.

### Questionnaire

Demographic and anthropometric data (age, gender, weight, education level, socioeconomic status, time from procedure), symptoms, and comorbidities were evaluated with questionnaires (the adjusted Gastrointestinal Symptoms Rating Scale Questionnaire and adjusted SIBO Questionnaire).

### Surgery and Histological Assessment

Bariatric surgery consisted of either RYGBP or LOAGBP, performed by the same surgeon (TP). Standard RYGBP procedure was performed with formation of 50-cm biliopancreatic limb and 100-cm jejunal exclusion with linear stapled anastomosis. For the LOAGBP, the procedure included a longer gastric pouch of 150 ml and 150 jejunal exclusion. Liver biopsy was performed and 1.5 cm^3^ of native liver tissue was analysed shortly after tissue retrieval.

One pathologist (BR), blinded to the patients’ clinical condition and biochemical data, evaluated every biopsy using NAFLD Brunt classification and NAFLD Activity Score (NAS) was calculated: steatosis was graded on a 0–3 scale, hepatocellular ballooning was graded on a 0–2 scale, lobular inflammation was graded on a 0–2 scale, and fibrosis was assessed on a five–stage scale. NASH was defined as NAS ≥ 5.

### Testing LMWH Application

All patients were tested to anti-Xa spontaneous values preoperatively and related to subtherapeutic LMWH application, tested 4 h after application, using the anti-Xa assay (chromogenic assay). The advantages of the test are that it is not affected by acute-phase reaction and is also unaffected by factor deficiencies, apart from antithrombin deficiency. One patient with antithrombin deficiency was excluded from the study.

### Statistical Analysis

Categorical variables were expressed as number of participants and percentage (%), numerical variables were expressed as mean value ± standard deviation, and in case of non-sufficient cohort number, a non-parametric test was used and in case of sufficient cohort number, parametrical Student’s *t* test was used for analysis. Nominal variables were analysed using the chi-squared test and the Fisher exact test, depending on sample size. The Spearman correlation was used for small observed group statistical analysis. The 95% confidence interval was calculated and a *p* value < 0.05 was considered statistically significant.

## Results

### Characteristics of the Population

Out of 56 patients after RYGBP/LOAGBP included in the study, 12 (21.4%) were men and 44 (78.6) were women, mean age was 49.54 ± 9.99 years (range 28–72 age), mean body weight was 88.700 ± 19.7523 kg (range 55.0–147.0 kg), mean BMI was 30.937 ± 6.55 kg/m^2^ (range 21.0–56.0 kg/m^2^), mean education level was 4.77 ± 1.32 (range 2–6), mean socioeconomic status was 5.84 ± 1.63 (range 2–9), and average time from surgery was 29.98 months (range 2–108 months).

### Incidence of SIBO and Factors Associated with SIBO

H_2_ glucose BT was positive in 24 (43%) and negative in 32 (57%) of patients. Characteristics of the patients according to positive or negative postoperative BT are presented at Table 1. Symptoms, comorbidities, and *Helicobacter pylori* infection according to positive or negative postoperative BT are presented at Table 2.

**Table 1:** Characteristics of patients according to positive or negative postoperative breath tests

Variables	Negative breath test (*n* = 32)	Positive breath test (*n* = 24)	*p*
Age (years)	49.5 ± 9.5	49.6 ± 10.8	0.842
Women, *n* (%)	25 (78.1%)	19 (79.2%)	1.00
BMI (kg/m^2^)	31.2 ± 6.8	30.5 ± 6.3	0.673
Education level	4.66 ± 1.31	4.92 ± 1.35	0.356
SE status	5.74 ± 1.79	5.96 ± 1.43	0.510
Time after S (months)	29.3 ± 15.8	30.9 ± 24.2	0.530

The variables are expressed as mean ± SD (standard deviation); *n* (%) represents the number and percentage of variable. Statistical analysis was done using Mann–Whitney *U* and Fisher exact tests; *p* < 0.05

*BMI*, body mass index; *S*, surgery; *SE*, socioeconomic status

**Table 2:** Symptoms, comorbidities, and *Helicobacter pylori* infection according to positive and negative postoperative breath test

Variable	Negative breath test (*n* = 32)	Positive breath test (*n* = 24)	*p*
Chronic pain	2.25 ± 1.85	2.38 ± 1.88	0.907
Diarrhoea	3.03 ± 2.21	2.96 ± 2.73	0.387
Frequent defecation	2.03 ± 1.00	2.58 ± 1.10	0.022*
Obstipation	1.78 ± 1.54	2.58 ± 2.45	0.356
Floating stools	1.56 ± 0.50	1.54 ± 0.51	0.878
Abdominal cramps	2.53 ± 1.85	2.96 ± 2.35	0.739
Flatulence and bloating	4.25 ± 2.45	5.71 ± 3.33	0.104
Nausea	2.00 ± 2.08	1.67 ± 1.40	0.596
Vomiting	1.38 ± 1.29	1.46 ± 1.06	0.585
Belching	3.28 ± 2.74	2.38 ± 1.79	0.357
Loss of appetite	1.34 ± 1.00	1.50 ± 1.14	0.429
Bloating	3.34 ± 2.27	3.96 ± 2.84	0.503
Fever	1.06 ± 0.25	1.17 ± 0.82	0766
Joint pain	2.81 ± 2.53	2.79 ± 2.08	0.765
Fatigue	3.19 ± 2.69	3.63 ± 2.63	0.461
Skin lesions	1.59 ± 1.60	2.88 ± 2.83	0.068
Confusion	2.00 ± 2.09	1.88 ± 1.60	0.670
Nausea with belching	2.84 ± 2.17	2.21 ± 1.50	0.347
Flatulence	2.63 ± 0.83	3.00 ± 1.06	0.135
Belching after meals	1.38 ± 1.16	1.83 ± 1.34	0.205
Pain and bloating	1.53 ± 1.08	1.54 ± 1.22	1.00
Obstipation	0.81 ± 1.23	1.08 ± 1.32	0.346
DO exchange	0.97 ± 1.15	0.92 ± 1.25	0.756
Diarrhoea	1.25 ± 1.16	1.08 ± 1.32	0.452
DO	0.75 ± 0.92	1.00 ± 1.41	0.835
Lactose intolerance	0.312 ± 0.940	0.688 ± 1.36	0.047*
Scleroderma	0.00 ± 0.00	0.06 ± 0.32	0.042*
Arthritis	1.07 ± 1.29	1.02 ± 1.31	0.815
Dermatitis	0.49 ± 1.04	0.50 ± 1.19	0.942
IBS	0.32 ± 0.89	0.75 ± 1.30	0.018*
Rosacea	0.06 ± 0.33	0.17 ± 0.63	0.147
Breathing problems	0.32 ± 0.83	0.33 ± 0.88	0.933
Headache	0.63 ± 0.90	0.71 ± 1.03	0.645
Memory loss	0.82 ± 1.14	0.98 ± 1.23	0.423
Diabetes	0.40 ± 1.15	1.13 ± 1.71	0.002*
*H. pylori*	1.63 ± 0.49	1.70 ± 0.47	0.618
*H. pylori* (*n* (%))	6 (30.0)	10 (37.0)	0.758

The variables are expressed as mean ± SD (standard deviation); *n* (%) represents the number and percentage of variable. Statistical analysis was done using Student’s *t* test and Mann–Whitney *U* test

^*^p < 0.05

DO, diarrhoea; obstipation; *IBS*, irritable bowel syndrome; *H. pylori*, *Helicobacter pylori*

### Liver Biochemistry and Histopathological Findings

Liver biochemistry values according to positive or negative postoperative BT are presented at Table 3. The histopathological findings according to positive or negative postoperative BT are presented at Table 4.

**Table 3:** Liver biochemistry according to positive and negative postoperative breath test

Variable	Negative breath test (*n* = 32)	Positive breath test (*n* = 24)	*p*
ALT surgery	0.592 ± 0.41	0.545 ± 0.23	0.802
ALT BT	0.522 ± 0.21	0.514 ± 0.33	0.266
AST surgery	0.516 ± 0.65	0.442 ± 0.16	0.352
AST BT	0.462 ± 0.15	0.428 ± 0.18	0.259

The variables are expressed as mean ± SD (standard deviation). Statistical analysis was done using Student’s *t* test

*ALT*, alanine aminotransferase; *AST*, aspartate aminotransferase; *BT*, breathing test

**Table 4:** Histopathological findings according to positive and negative postoperative breath test

Variable	Negative breath test (*n* = 32)	Positive breath test (*n* = 24)	*p*
NAS	3.00 ± 1.80	3.33 ± 1.78	0.594
Steatosis	1.44 ± 0.89	1.56 ± 0.86	0.680
Lobular inflammation	0.741 ± 0.594	0.889 ± 0.583	0.402
HB	0.815 ± 0.623	0.889 ± 0.583	0.666
Fibrosis	0.481 ± 0.509	0.294 ± 0.470	0.220
Fibrosis	51.9%	70.6%	0.346
NASH (*n* (%))	5 (18.5%)	4 (22.2%)	1.00

The variables are expressed as mean ± SD (standard deviation); *n* (%) represents the number and percentage of variable. Statistical analysis was done using the Mann–Whitney *U* test and Fisher’s exact test

*HB*, hepatocellular ballooning; *NAS*, NAFLD Activity Score; *NASH*, non-alcoholic steatohepatitis

In higher NAS, higher difference between anti-Xa and reference range was calculated; we also calculated positive and important correlation to other systemic inflammatory parameters (SIBO — peridontal disease related to NAS; the higher the grade of parodontitis, the higer the difference to range value before and after LMWH application).

**Fig. 1:**
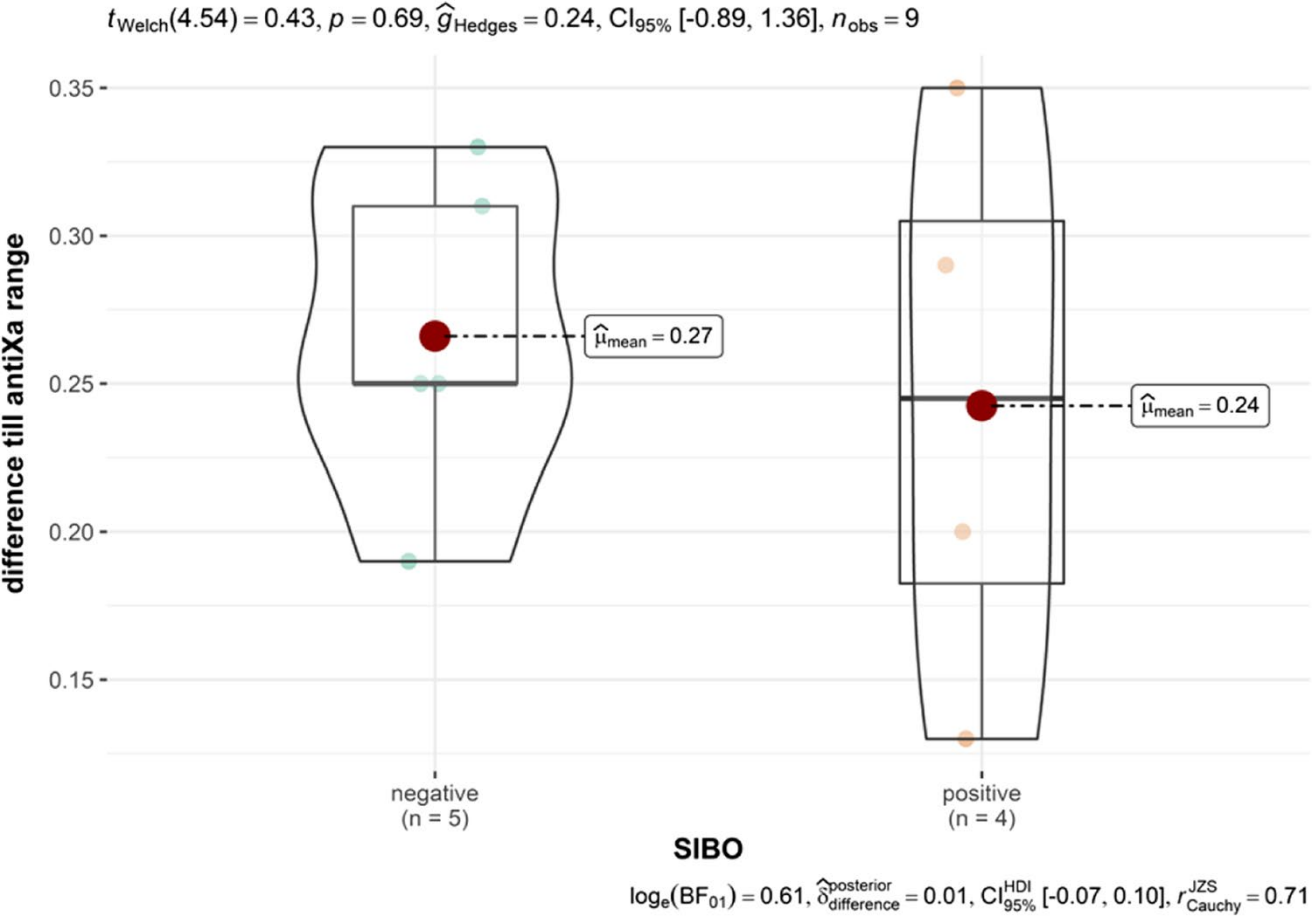
Difference till anti-Xa range in SIBO-positive and SIBO-negative group before LMWH application

**Fig. 2:**
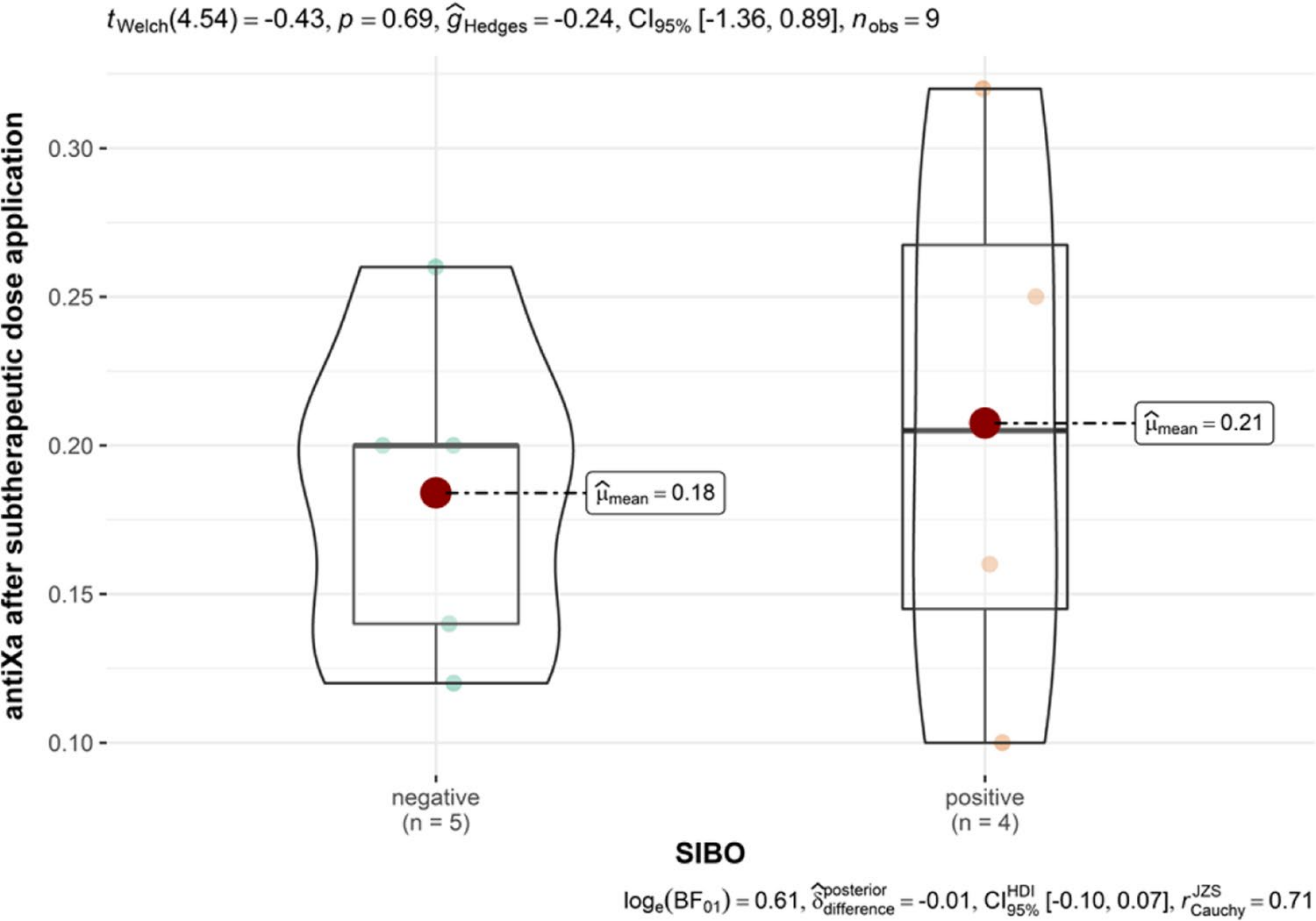
Difference till average value in SIBO-positive and SIBO-negative group. There is a positive statistical trend in calculated difference between observed values and average values after subtherapeutic LMWH application. In the SIBO-positive group, lower values of anti-Xa were measured and there is a greater difference to the cross-sectional value of anti-Xa 4 h after subtherapeutic dose application

NAS correlated with BMI, diabetes, HDL, glucose levels, SIBO-positive patients, and difference till anti-Xa range 4 h after subtherapeutic LMWH application (Figs. 1 and 2, Table 5).

**Table 5:** Trying kwt

	1 (*n* = 6)	2 (*n* = 10)	3 (*n* = 13)	*p*
Anti-Xa BA				0.314
Mean (SD)	0.0133 (0.0103)	0.0250 (0.0237)	0.0108 (0.0144)	
Median (Q1, Q3)	0.0200 (0.0050, 0.0200)	0.0300 (0.0000, 0.0400)	0.0000 (0.0000, 0.0200)	
Min–max	0.0000–0.0200	0.0000–0.0600	0.0000–0.0400	
Missing values	0	0	0	
Anti-Xa SDA				0.344
Mean (SD)	0.2317 (0.0906)	0.1920 (0.0699)	0.1682 (0.0710)	
Median (Q1, Q3)	0.2350 (0.1625, 0.2925)	0.2000 (0.1250, 0.2375)	0.1400 (0.1200, 0.1600)	
Min–max	0.1200–0.3500	0.1000–0.3100	0.1060–0.3200	
Missing values	0	0	0	
DTanti-Xa range				0.344
Mean (SD)	0.2183 (0.0906)	0.2580 (0.0699)	0.2818 (0.0710)	
Median (Q1, Q3)	0.2150 (0.1575, 0.2875)	0.2500 (0.2125, 0.3250)	0.3100 (0.2900, 0.3300)	
Min–max	0.1000–0.3300	0.1400–0.3500	0.1300–0.3440	
Missing values	0	0	0	

*BA*, before application; *DT*, difference till; *SDA*, after subtherapeutic dose application

## Discussion

Human gut microbiota is composed of a mosaic of microorganisms that vary between individuals, which represents personalized microbiological identity [6]. More than 90% of the total microbial population in humans are dominated by four bacterial phyla: *Firmicutes* (40%), *Bacteroidetes* (19.7%), *Actinobacteria* (2.15%), and *Proteobacteria* (2.15%) [3, 14–16]. The quantitative and qualitative imbalance between these phyla, which occurs in SIBO, seems to be the key to the origin of various pathologies [6, 10, 17–23].

Modification of normal gut anatomy after bypass bariatric procedures with procedure being restrictive, malabsorptive, and creating an excluded blind loop, is creating a suitable environment for bacterial stasis and consequent development of SIBO [2]. In a recent study published by Bastos et al., it has been indicated that the shortening of gut itself, and not the blind loop, was the major factor for SIBO [24].

The present study was the first to evaluate the incidence of SIBO after two different bypass bariatric procedures in symptomatic and asymptomatic patients, presenting an increased incidence of SIBO, being 43%. Sabate et al. and Coelho et al. found similar incidence of SIBO but only after RYGBP, 40% and 38.8% respectively, but Coelho et al. did not report if the patients were asymptomatic, symptomatic, or both. Also, the studies do not offer a detailed description of the operations, especially the length of exclusion of the alimentary limb and the creation of anastomoses that might be an important contributing factor to SIBO. As one of the predisposing factors is also nutritional treatment, data would be mandatory for further discussion. A recent study found 83% incidence of SIBO after RYGBP, OAGBP, and sleeve gastrectomy in symptomatic patients [5]. These studies are difficult to compare, because of different modalities of glucose substrates used (25–75 g). Regarding the high incidence of SIBO in asymptomatic and symptomatic patients, it would be reasonable to test asymptomatic patients as well.

In the present study, SIBO was associated with different comorbidities, such as scleroderma, irritable bowel syndrome, diabetes, and lactose intolerance, which are common diseases coexisting with SIBO [18, 19, 25–27]. Demographic and anthropometric data were not associated with SIBO which is consistent with most of the literature; to the best of our knowledge, only two studies found association with female sex [2, 5] and one study increased incidence with advanced age [5].

In our study, we found higher frequency of defecation in patients with SIBO, but despite that, no single clinical sign or symptom nor their intensity or frequency was connected with SIBO-positive test. Bloating is considered the most common symptom related to SIBO [13] and along with abdominal pain, flatulence, and diarrhoea, these symptoms can be consistent with SIBO but are non-specific [2]. The diagnosis of SIBO cannot be set based only on clinical signs or symptoms, therefore [2, 18], and effective diagnostic method seems to be H_2_BT, which are non-invasive, precise, and useful for diagnosing SIBO, although the lack of standardized methodology remains problematic.

In patients with obesity, changes in dietary composition have also been associated with changes in the composition of the gut microbiota, especially increased *Firmicutes/Bacteroidetes* ratio, according to most of the studies [1, 17, 28]. Changed composition of gut microbiota, also characteristic in the case of SIBO, is connected with a higher capacity to extract energy from the diet by providing more enzymes for the breakdown of dietary polysaccharides, thereby increasing the uptake of monosaccharide and short-chain fatty acids (SCFA) by the intestinal mucosa [29]. An energy-balanced study revealed that a 20% increase in *Firmicutes* and 20% decrease in *Bacteroidetes* were associated with an additional energy harvest of 150 kcal per day. In patients with obesity, changes in gut-microbiota brain axis are also present and are possibly connected to preference in certain (more caloric) food pattern and positive energy balance in these patients [23]. Based on these findings, SIBO could be involved in weight regain, i.e. a lower effect of bariatric surgery treatment.

The fasting or postprandial total bile acid (BA) concentrations are increased after RYGBP and LOAGBP procedure, which is beneficial to weight loss, because of their action through farnesoid X receptor (FXR) and membrane Takeda G protein–coupled receptor 5 (TGR5) in the regulation of lipid accumulation and gluconeogenesis as FXR and TGR5 agonists decrease lipogenesis, improve hypercholesterolemia, increase energy consumption, and decrease systemic inflammation [30]. In the case of SIBO after bariatric surgery, a beneficial effect of BA is lost [30]. Another important characteristic in patients with morbid obesity is systemic low-grade inflammation, which is possibly due to metabolic endotoxemia because of SIBO [31]. Different studies have proved systemic endotoxemia involvement in different complications, such as thromboembolism, systemic and skin inflammatory diseases, and different types of cancer [31]. With previously described mechanisms, we could partly explain insufficient weight loss in certain patients after RYGBP/LOAGBP and development of different metabolic and systemic complications.

Study results confirmed higher baseline spontaneous values of anti-Xa in SIBO-positive patients and importantly lower measured values after subtherapeutic LMWH application till range average value with important statistical trend, presented with the Spearman correlation for small group analysis. This important result dictates the continuation of the study and the search for a causal relationship between the systemic inflammatory response, SIBO, and the contribution of other vent events in morbidly obese subjects. For the same group of observed patents, Čolak et al. presented important correlation to periodontal disease and NAS that have been also calculated in SIBO-positive patients [9]. The result means that in patients with a higher NAS, we note the presence of a higher level of periodontal inflammation and, at the same time, a reduced response to the application of a subtherapeutic dose of LMWH with a no deviation of liver test values tested SIBO only but tested SIBO and periodontal disease a correlation to liver test (AST and ALT) was calculated and statistically important. These findings are clinically relevant and confirm our assumptions that compensatory physiological mechanisms maintain relatively normal values of liver function tests despite the impaired liver function that the statistical analysis of our data shows.

We are aware that in order to confirm our results, a more comprehensive study is necessary, which could use these results as preliminary and, on the basis of the latter, determine a set of data to search for connections and responsible mechanisms of this phenomenon. Literature search did not present any similar study up to now.

In the present study, we found higher tendency of liver damage in patients with SIBO condition after RYGBP/LOAGBP, based on higher values of NAS in SIBO-positive patients with higher values also in separate spectres of NAS (steatosis, portal inflammation, hepatocellular ballooning) and higher frequency of NASH and fibrosis, although values were not statistically significant. Mikolasevic et al., like our study, found higher values in patients with SIBO, but with statistically significant values on patients, but they did not test patients after bariatric procedures [32]. One possible reason we did not find statistically significant values is that in our study we tested patients in average 29 months after RYGBP/LOAGBP and with bariatric surgery after 6 months improved NAS, liver enzyme values as well as systemic inflammation along with body weight reduction are seen, which is favourable for improvement of the liver damage. We also found discrete correction of liver enzyme values (AST and ALT) after RYGBP and OAGBP in patients with SIBO in comparison to patients without SIBO.

Our results are suggesting SIBO involvement in the pathophysiology of NAFLD, i.e. MAFLD and severity of liver injury. Anatomical and functional relationship between the digestive tract and liver ensures a theoretical hypothesis that liver acts as a target of gut microbiota [32] and other focuses of inflammation, as presented in our study, periodontal disease. There is increasing evidence of correlation between gut microbiota dysbiosis and MAFLD as well as its severity and complications (ascites, encephalopathy, bacterial peritonitis, and portal hypertension) [12, 32] and correlation to other inflammatory focuses, also. In the case of SIBO, gut barrier permeability is increased, which promotes translocation of bacterial toxins and its products (especially lipopolysaccharide (LPS)). LPS is likely to activate Toll-like receptor 4 (TLR-4), a CD-14 receptor, by stimulating expression of nuclear factor kappa B (NF-κB) leading to increase in production of certain proinflammatory cytokines, such tumour necrosis factor α (TNF-α), interleukin-1β (IL-1β), interleukin-6 (IL-6), and interleukin-8 (IL-8) [32, 33]. Excessive production of these cytokines induces the development of inflammation as well as insulin resistance and is considered significant in NASH, liver fibrosis, and HCC pathogenesis [32].

There are few limitations to this study. The first limitation relates to the relatively small number of participants who had undergone two different surgical procedures in respect to equal length of small intestinal exclusion. The second limitation is that BT may be problematic to diagnose SIBO following RYGBP and LOAGBP, as rapid transit through small bowel could result in the test substrate (in our case glucose) reaching the colon and initiating fermentation by colonic bacteria, leading to an early rise in breath hydrogen that might be falsely attributed to bacteria in the small bowel [6]. The third limitation is use of 25 g of glucose, instead of 75 g, which could be the reason for underestimating the incidence of SIBO in our study. Final limitation is not evaluating the incidence of SIBO before bariatric surgery procedures; as we know in patients with morbid obesity, prevalence of SIBO is higher than in healthy population. We understand that we could influence the incidence of SIBO with dietary supplementation and treatment and with mandatory preoperative eradication of *Helicobacter pylori* infection, both of which could lower the real incidence.

## Conclusion

The incidence of SIBO after RYGBP and LOAGBP is 43%, which is alarmingly high, and the diagnosis cannot be made based on specific symptom or their intensity. The presence of SIBO is currently underestimated and often misdiagnosed, which is associated with the occurrence of systemic metabolic complications as well as with negative systemic influence of the changed gut microbiota. The results of our study confirmed a correlation of SIBO to NAS, reduced response to LMWH application and increased anti-Xa baseline values and a positive correlation to SIBO, periodontal disease and NAS and deviation from the transverse values of liver tests. The results of our study confirm that there is an urgent need to systematically address SIBO after such surgical procedures, thereby affecting the clinical outcome of the underlying disease, improving it significantly and preventing the development of complications. More robust results would be needed to demonstrate an effect on liver synthetic function that was demonstrated negative in SIBO-positive patients.
